# Simultaneous *CXCL12 *and *ESR1 *CpG island hypermethylation correlates with poor prognosis in sporadic breast cancer

**DOI:** 10.1186/1471-2407-10-23

**Published:** 2010-01-28

**Authors:** Edneia AS Ramos, Anamaria A Camargo, Karin Braun, Renata Slowik, Iglenir J Cavalli, Enilze MSF Ribeiro, Fábio de O Pedrosa, Emanuel M de Souza, Fabrício F Costa, Giseli Klassen

**Affiliations:** 1 Department of Basic Pathology, Federal University of Parana, Curitiba, Brazil; 2 Laboratory of Molecular Biology and Genomics, Ludwig Institute for Cancer Research, SP, Brazil; 3 Department of Genetics, Federal University of Parana, Curitiba, Brazil; 4 Department of Biochemistry and Molecular Biology, Federal University of Parana, Curitiba, Brazil; 5 Cancer Biology and Epigenomics Program, Children's Memorial Research Center and Northwestern University's Feinberg School of Medicine, Chicago, USA

## Abstract

**Background:**

CXCL12 is a chemokine that is constitutively expressed in many organs and tissues. *CXCL12 *promoter hypermethylation has been detected in primary breast tumours and contributes to their metastatic potential. It has been shown that the oestrogen receptor α (*ESR1*) gene can also be silenced by DNA methylation. In this study, we used methylation-specific PCR (MSP) to analyse the methylation status in two regions of the *CXCL12 *promoter and *ESR1 *in tumour cell lines and in primary breast tumour samples, and correlated our results with clinicopathological data.

**Methods:**

First, we analysed *CXCL12 *expression in breast tumour cell lines by RT-PCR. We also used 5-aza-2'-deoxycytidine (5-aza-CdR) treatment and DNA bisulphite sequencing to study the promoter methylation for a specific region of *CXCL12 *in breast tumour cell lines. We evaluated *CXCL12 *and *ESR1 *methylation in primary tumour samples by methylation-specific PCR (MSP). Finally, promoter hypermethylation of these genes was analysed using Fisher's exact test and correlated with clinicopathological data using the Chi square test, Kaplan-Meier survival analysis and Cox regression analysis.

**Results:**

*CXCL12 *promoter hypermethylation in the first region (island 2) and second region (island 4) was correlated with lack of expression of the gene in tumour cell lines. In the primary tumours, island 2 was hypermethylated in 14.5% of the samples and island 4 was hypermethylated in 54% of the samples. The *ESR1 *promoter was hypermethylated in 41% of breast tumour samples. In addition, the levels of ERα protein expression diminished with increased frequency of *ESR1 *methylation (p < 0.0001). This study also demonstrated that *CXCL12 *island 4 and *ESR1 *methylation occur simultaneously at a high frequency (p = 0.0220).

**Conclusions:**

This is the first study showing a simultaneous involvement of epigenetic regulation for both *CXCL12 *and *ESR1 *genes in Brazilian women. The methylation status of both genes was significantly correlated with histologically advanced disease, the presence of metastases and death. Therefore, the methylation pattern of these genes could be used as a molecular marker for the prediction of breast cancer outcome.

## Background

Breast cancer development and progression is influenced by intrinsic properties of the tumour cells, as well as by macro-environmental factors. There is an extensive interplay between tumour cells and signalling molecules such as chemokines [1,2]. Chemokine receptors and growth factors have been extensively implicated in the metastatic process of breast cancer [3]. A chemokine-mediated process of tumour cell homing to specific metastatic sites requires an enrichment in the site of metastasis formation for specific chemokines; these chemokines are then able to induce the migration of tumour cells that express the corresponding receptors [3]. CXCL12, formerly known as stromal cell-derived factor-1 (SDF-1α), is a CXC subfamily of chemokines that is expressed by stromal cells, including fibroblasts and endothelial cells. CXCL12 is also known to be present in the organs that are target for metastasis in breast cancer [1].

In cancer cells, gene expression is commonly altered due to a combination of genetic and epigenetic events. Aberrant gene silencing in mammalian cells is associated with promoter methylation, and it is known that many regions of the genome are methylated at one or more CpG sites [4,5]. Recent studies have also demonstrated that the *CXCL12 *gene modulates metastatic potential in breast and colon carcinomas, where it controls its own regulation in an autocrine loop. Epigenetic silencing causes the loss of autocrine expression and results in an imbalance in the expression levels of CXCL12 and its receptor, CXCR4 [6,7].

Sixty percent of primary breast tumours are ERα-positive, and two-thirds of advanced breast tumours respond to therapy with anti-estrogens such as tamoxifen (Novaldex^®^) [8]. However, a fraction of tumours that are ERα-positive at diagnosis subsequently lose ERα expression during the progression of the disease [9]. Hypermethylation of the oestrogen receptor α gene (*ESR1*) is a common occurrence in several specific populations and for workers in a number of occupations; it seems to be a relevant factor for hormonal treatment [10,11]. In this study, we evaluated the methylation patterns of *ESR1 *and two CpG islands in the *CXCL12 *gene in breast tumour samples from Brazilian women. This is the first study to report an association between simultaneous DNA methylation of these two genes compared to other prognostic factors in breast cancer among Brazilian women.

## Methods

### Cell Lines

Breast tumour cell lines were all obtained from the Ludwig Institute for Cancer Research, (São Paulo, Brazil). The following cell lines were used: MDA-MB-436, MDA-MB-435, MDA-MB-231, MCF-7, PMC42, HB4a (control immortalized normal cells) [12] and HB4aC3.6 [13]. The cell lines were cultured in RPMI 1640 medium [14] containing 10% foetal bovine serum (supplemented with 0.2 mM glutamine, and 40 μg/mL garamycin, 10 μg/mL insulin, if necessary) at 37°C in a humidified incubator with 5% CO_2_.

### Patient samples

Frozen samples of breast tumours (n = 69) used for methylation analysis were obtained from patients treated by primary surgery for breast cancer at the Nossa Senhora das Graças Hospital, Curitiba, PR, Brazil with institutional approval (Process number 25000.007020/2003-93; CONEP register 7220 opinion number 251/2003). The study included only female patients with invasive breast tumours. All patients gave informed consent for the study to retain and analyse their tissue for research purposes. The ages of the patients ranged from 27 to 84 years (mean 57.8 ± 14.7). Histological types were either infiltrative ductal carcinoma (IDC) (n = 51, 73.9%) or infiltrative lobular carcinoma (ILC) (n = 18, 26.1%). The lymph node status of the patients was positive (n = 35, 51.5%) or negative (n = 33, 48.5%). Histologic grade was determined according to the modified Bloom-Richardson criteria. Of the patients, 27.6% were Grade I, 47.8% were Grade II and 24.6% were Grade III. TNM staging was done according to official classification methods [15]. Other clinicopathological data (tumour size, local recurrence, metastasis and death) are summarised in Table 1.

**Table 1:** Clinicopathological features of the 69 patients with primary breast carcinomas according to methylation status of *CXCL12 *and *ESR1 *genes

**Variables**	**Samples (%)**	***CXCL12 *methylation**^**(a)**^		***p***^***a***^	***CXCL12 *methylation**^**(b)**^		***p***^***b***^	***ESR1 *methylation**^**(c)**^		***p***^***c***^
				
		**M (%)**	**U (%)**		**M (%)**	**U (%)**		**M (%)**	**U (%)**	
							
**Age**										
< 45	9 (13)	0	9 (100)	0.3378	3 (33)	6 (67)	0.2853	2 (22)	7 (78)	0.2937
≥ 45	60 (87)	10 (17)	50 (83)		34 (56.7)	26 (43.3)		26 (43)	34 (57)	
**Stage**										
I	13 (19.7)	2 (15.4)	11 (84.6)		5 (38.5)	8 (61.5)		1 (7.7)	12 (92.3)	
II	31 (47)	5 (16.1)	26 (83.9)	0.9691	13 (42)	18 (58)	0.3696	10 (32.3)	21 (67.7)	**0.0003**
III/IV	22 (33.3)	3 (14)	19 (86)		13 (59.1)	9 (40.9)		16 (72.7)	6 (27.3)	
**Tumour size**										
pT1	19 (27.6)	4 (21)	15 (79)		10 (53)	9 (47)		4 (21)	15 (79)	
pT2	36 (52.2)	4 (11.1)	32 (88.9)	0.6088	17 (47.2)	19 (52.8)	0.3034	13 (36.1)	23 (63.9)	**0.0029**
pT3/pT4	14 (20.2)	2 (14.3)	12 (85.7)		10 (71.4)	4 (28.6)		11 (78.6)	3 (21.4)	
**SBR**										
I	19 (27.6)	4 (21.1)	15 (78.9)		7 (36.8)	12 (63.2)		2 (11)	17 (89)	
II	33 (47.8)	4 (12)	29 (88)	0.4178	17 (39.4)	16 (60.6)	**0.0180**	14 (42.4)	17 (57.6)	**0.0011**
III	17 (24.6)	2 (11.8)	15 (88.2)		13 (65)	4 (35)		12 (71)	5 (29)	
**Lymph node status**										
Positive	35 (51.5)	5 (14.3)	30 (85.7)	1.0000	22 (63)	13 (37)	0.2231	19 (54.3)	16 (45.7)	**0.0288**
Negative	33 (48.5)	5 (15.1)	28 (84.9)		15 (45.4)	18 (54.6)		9 (27.3)	24 (72.7)	
**Estrogen receptor**										
Positive	57 (83.8)	7 (12.2)	50 (87.8)	0.6312	29 (51)	28 (49)	0.2084	19 (33)	38 (67)	**0.0054**
Negative	11 (16.2)	2 (18.2)	9 (81.8)		8 (73)	3 (27)		9 (81.8)	2 (18.2)	
**HER2**										
Positive	20 (31.3)	5 (25)	15 (75)	0.0955	12 (60)	8 (40)	0.5996	10 (50)	10 (50)	0.4249
Negative	44 (68.7)	3 (6.9)	41 (93.1)		23 (52.3)	21 (47.7)		17 (38.6)	27 (61.4)	
**Progesterone receptor**										
Positive	45 (65)	7 (17)	38 (83)	1.0000	25 (56)	20 (44)	1.0000	16 (36)	29 (64)	0.0808
Negative	16 (35)	2 (12.5)	14 (87.5)		9 (56.3)	7 (43.7)		10 (62.5)	6 (37.5)	
**Metastasis**										
Positive	15 (22.4)	3 (20)	12 (80)	0.4075	14 (93)	1 (7)	**0.0008**	13 (86.7)	2 (13.3)	**< 0.0001**
Negative	52 (77.6)	6 (11.5)	46 (88.5)		23 (44)	29 (56)		14 (26.9)	38 (73.1)	
**Recurrence**										
Positive	8 (12)	2 (25)	6 (75)	0.3413	6 (75)	2 (25)	0.2700	4 (50)	4 (50)	0.7049
Negative	59 (88)	8 (13.5)	51 (86.5)		30 (51)	29 (49)		23 (39)	36 (61)	
**Death**										
Positive	17 (26)	4 (23.5)	13 (76.5)	0.2250	15 (88)	2 (12)	**0.0019**	13 (76.5)	4 (23.5)	**0.0013**
Negative	48 (74)	5 (10.4)	43 (89.6)		21 (43.7)	27 (56.3)		14 (37.8)	33 (62.2)	
**Histological type**										
IDC	51 (73.9)	8 (15.7)	43 (84.3)	1.0000	30 (58.8)	21 (41.2)	0.1759	20 (39.2)	31 (60.8)	0.7829
ILC	18 (26.1)	2 (11.1)	16 (88.9)		7 (39)	11 (61)		8 (44.4)	10 (55.6)	

Abbreviations: a, data of *CXCL12 *CpG island-2; b, data of *CXCL12 *CpG island-4; c, data of *ESR1 *gene; *p*, value from statistical analysis χ^2 ^test and Fisher's exact test; M, methylation results; U, unmethylation results; significant data are in bold.

### Immunohistochemistry

Standard immunohistochemical (IHC) detection was performed on sections from archival paraffin embedded breast tumour tissues. Protein expression in malignant breast tissues was detected with specific antibodies against estrogen receptor (ER) and progesterone receptor (PR); clones 1D5 and PgR 636 (DAKO), respectively. Monoclonal mouse anti-human antibodies were pre-diluted and incubated for 18 h at 4°C according to the manufacturer's instructions. The rabbit polyclonal antibody against HER2 detection was performed by the HercepTest™ (DAKO CYTOMATION code K5204). In addition, positive and negative controls for each marker were routinely performed during experiments.

Sections were then processed using the EnVision™FLEX Target Retrieval Solution (DAKO) according to the manufacture's recommendations. Immunohistochemical staining of the samples was evaluated and scored by two pathologists who were responsible for clinicopathological data. The cutt-of value for ER and PR status values was 10% of cells. Fifty-seven ERα-positive tumours were scored based on the number of positive cells present in IHC staining. The slides were scored following the criteria: <10% tumour cells showing nuclear staining scored as 0, 10-30% tumour cells showing nuclear staining scored as 1, 30-50% tumour cells showing nuclear staining scored as 2, >50% tumour cells showing nuclear staining scored as 3.

### RNA extraction and reverse transcription

Total RNA was isolated using the TRIzol Reagent (Life Technologies, USA) according to the protocol supplied by the manufacturer. Reverse transcription reactions were performed using 500 ng of DNA-free RNA, an oligo (dT)_12-18 _primer and Superscript II Reverse Transcriptase (Gibco, BRL). PCR was performed using *CXCL12*-specific primers, and *GAPDH *was used as a housekeeping control (Table 2). The PCR was performed in a volume of 20 μl containing 1× PCR buffer (Invitrogen), 1.5 mM MgCl_2 _(Invitrogen), 200 μM dNTPs, 0.3 μM of each primer and 1 U of T*aq *Platinum (Invitrogen). The PCR conditions were as follows: 95°C for 10 min, 94°C for 45 s, the appropriate annealing temperature for 45 s, 72°C for 1 min and a final extension of 72°C for 5 min. PCR products were resolved on 1% agarose gels and visualised by ethidium bromide staining.

**Table 2:** Summary of primer sequences, used for RT-PCR, nested-PCR and MSP

Application and specificity	CpG status	Forward primer (5'-3')	Reverse primer (5'-3')	Product size (bp)	Annealing T (°C)	**Ref**.
**RT-PCR**						
*CXCL12*	-	CAACGTCAAGCATCTCAA	AGCTGCAATATCATACCGTA	383	58	This work
*GAPDH*	-	CTGCACCACCAACTGCTT A	CATGACGGCAGGTCAGGTC	296	63	
**nested-PCR**						
*CXCL12*	-	GTAGTGAGGTTTAGTGAAG	CCATAAATACCACAATAACTTC	479	51, 53, 55	This work
*CXCL12*-nested	-	AGGTTTTTGTTGGGTTGG	CAAATCCTAAATCCAACTAC	338	53, 55, 57	
SATR-1	-	GTTATATTATTTTTTGTTTTTTTG	ACATTTCCTTATAATATTATTCC	-	48, 50, 52	[21]
SATR-1 nested	-	TATAGTGGTGGTGTATATTTG	CACCTAACCTATAATATTTCTTC	690	52, 54, 56	
**MSP**						
*CXCL12***- (2)**	**U**	GAGTTTGAGAAGGTTAAAGGTTGG	CAAAAAATAAAAATACAACA	249	50	[7]
*CXCL12***- (2)**	**M**	GTTAAAGGTCGGAGCGTATTG	ACGAAAAATAAAAATACGACGAT	237	59.5	
*CXCL12***- (4)**	**U**	GTCGTAGCGTTGGGGTT	AACGAAACTACGCGCGACT	200	57.8	This work
*CXCL12***- (4)**	**M**	GTTGTAGTGTTGGGGTTT	AAACAAACAAAACTACA	189	64	
*ESR1*	**M**	ACGAGTTTAACGTCGCGGTC	ACCCCCCAAACCGTTAAAAC	140	57.6	[20]

Abbreviations: M, specific for methylated condition; U, specific for unmethylated condition; (2), primers designed for CpG island 2; (4), primers designed for CpG island 4; *ESR1*, estrogen receptor gene.

### 5-aza-2'-deoxycytidine (5-aza-CdR) treatment

The cell lines MDA-MB-231, MDA-MB-435 and MDA-MB-436 were analysed using this technique. Cells were plated (10^6 ^cells/ml) and treated for 7 days with 1 μM 5-aza-CdR (Sigma Aldrich, Deisenhein, Germany) or left untreated. The medium was changed every day, and no significant cell death was observed. After 7 days of treatment, total RNA was isolated. The expression of *CXCL12 *in breast tumour cells was analysed by RT-PCR using the housekeeping gene *GAPDH *as an internal control. PCR products were resolved on 1% agarose gels and visualised by ethidium bromide staining.

### DNA isolation and sodium bisulphite treatment

Genomic DNA was prepared from breast cancer cell lines or frozen tumour samples by the phenol/chloroform protocol [16]. They were then subjected to sodium bisulphite treatment using the EpiTect^® ^Bisulphite Kit (Qiagen) according to the manufacturer's instructions.

### CXCL12 CpG island methylation analysis

Previous studies used the MSP technique to evaluate the regulation of *CXCL12 *expression by DNA methylation and in this study we have utilized the designed primers for this region (island 2), as described [7].

**Figure 1:**
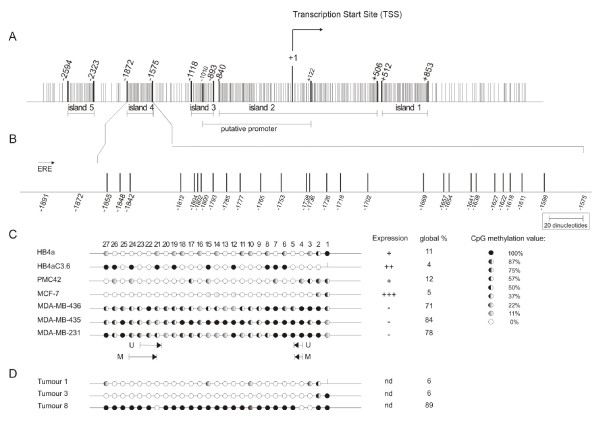
**The CpG island of the *CXCL12 *gene**. **(A) **The CpG island is inside an area from -2594 to +853 (data of the CpGPlot program). The vertical lines correspond to the CpG dinucleotides. The numbers above correspond to the distance in relation to the +1 (TSS). The area considered with promoter activity is outlined and it corresponds to the position -1010 to +122 [22]. **(B) **Twenty-seven dinucleotides are represented in the figure and positioned in scale. Its localization is signaled below in the figure and the distance in relation to the +1 (TSS). The ERE factor binding is located 19 nucleotides upstream to the CpG island 4 in nucleotides -1900 to -1918. **(C) **Twenty-seven dinucleotides are numbered in agreement with the sequence. The open circles represent the unmethylated dinucleotides while the dark portion represents the percentage of methylation. On the right side methylation pattern are represented according to data of RT-PCR and the absolute percentage value. The MSP primers for the condition M (methylated) and U (unmethylated) for this island are located below in the figure. **(D) **Representative examples of sequenced tumours. On the right the methylation percentage of three primary tumours is represented. Global % is the number of methylated CpGs divided by the total analyzed.

In an attempt to determine all CpG islands and all potential transcription start sites (TSS) for *CXCL12*, we first proceeded with the identification of the promoter sequence [17]. The analyses were initiated using an identified RefSeq by GenBank accession number, after which we submitted the gene sequence to a Genome BLAT Search through the UCSC Genome bioinformatics website http://genome.ucsc.edu. We selected 2000 bps of sequence extending from the 5' upstream region to 1000 bps downstream of the region of the TSS. The BLAT program returned a sequence of 5677 bps that was first submitted to the CpGPLOT program from the European Bioinformatics Institute website http://www.ebi.ac.uk/emboss/cpgplot. This program defines a CpG island as ≥ 200 bps of sequence with ≥ 50% C + G content and ≥ 0.6 CpG observed/CpG expected. The 5677 bps from *CXCL12 *that we have analysed contained five CpG islands in a region of 3447 bps (Figure 1A). The 5677 bps sequence was also submitted to computational analysis to predict transcription factor binding sites using TESS http://www.cbil.upenn.edu/cgi-bin/tess/tess and MatInspector http://www.genomatix.de/[18]. The DNA region we refer to as island 4 (Figure 1A) is positioned next to an estrogen responsive element (ERE) binding site that could be involved in breast cancer. This CpG island was also selected for methylation status analyses in this study.

Island 4 was amplified from bisulphite-treated DNA samples using a nested-PCR amplification protocol. The two sets of primers were used for the nested reactions at their appropriate annealing temperatures, and are shown in Table 2. The first PCR reactions were performed as described below: 1 cycle of 95°C for 10 min, 94°C for 3 min, the appropriate annealing temperature for 3 min, 72°C for 2 min; 5 cycles of 94°C for 3 min, annealing temperature for 3 min, 72°C for 2 min; 35 cycles of 94°C for 1 min, annealing temperature for 1 min, and 72°C for 5 min. Amplified products were purified using the Qiaquick Gel Extraction Kit (Qiagen) and cloned into a pCR2.1 cloning vector (Invitrogen). Eight clones were sequenced for each cell line using the universal or reverse primers. DNA sequencing reactions were performed using Big Dye Terminator technology (Applied Biosystems) on an ABI 377 sequencer (Applied Biosystems) according to the manufacturer's instructions. One hundred percent methylation was obtained if a methylated cytosine in the CpG dinucleotides was present in eight sequenced clones. The methylation percentage for each tumour cell line (global methylation pattern) was calculated by dividing the number of methylated CpG dinucleotides by the total number of CpGs analysed.

### Methylation-specific PCR (MSP)

Methylation-specific PCR was performed as previously described [19] and the *CXCL12 *primer sequences for island 2 [7] can be visualized in the Table 2. gDNA from the primary breast tumours was bisulphite-modified and amplified with *CXCL12 *primers specific for methylated (M) and unmethylated (U) DNA. Analysis of methylation of the *ESR1 *promoter was performed using the set of primers described by Li et al. [20]. PCR reactions were performed with 1 μL of modified DNA, 1× PCR Buffer (Invitrogen), 1.5 mM (for *CXCL12*) or 2.5 mM (for *ESR1*) of MgCl_2 _(Invitrogen), 200 μM dNTPs, 0.3 μM of each primer and 1 U of T*aq *Platinum (Invitrogen). The PCR protocol was 95°C for 10 min; 38 cycles of 94°C for 45 s, the appropriate annealing temperature for 30 s and 72°C for 45 s, followed by a final extension of 72°C for 5 min. DNA modification was confirmed by a nested-PCR reaction with a set of primers for a previously described satellite region (Table 2). This reaction was used as a control for bisulphite modification quality [21]. This PCR reaction, the nested-PCR and temperature conditions are described in Table 2. The amplification products were separated on 2% agarose gels and stained with ethidium bromide.

### Statistical analysis

The statistical analysis was carried out using SPSS program (version 16.0, SPSS Inc., *Chicago, Illinois, USA*). Associations between specific clinicopathological parameters were analyzed using Chi square test and Fisher's exact test. Statistical significance was assumed for *p *< 0.05. The overall survival was calculated from the time of diagnoses of disease to the occurrence of death. Survival data were censored on 30^th ^June, 2009, which was the date in which the survival data were correlated with the death registry for the last time of a means of 91 months after onset of the disease. Kaplan-Meier estimates are presented for the survival functions, and differences in survival were analyzed using the log rank test. Multivariate analysis was conducted with a backward application of Cox proportional hazards regression analysis was used to estimate hazards ratio (HR) and 95% confidence intervals (95% CI) for overall survival and metastasis-free survival. All covariates with p < 0.25 were retained in the final model.

## Results

### CXCL12 expression in breast tumour cell lines

**Figure 2:**
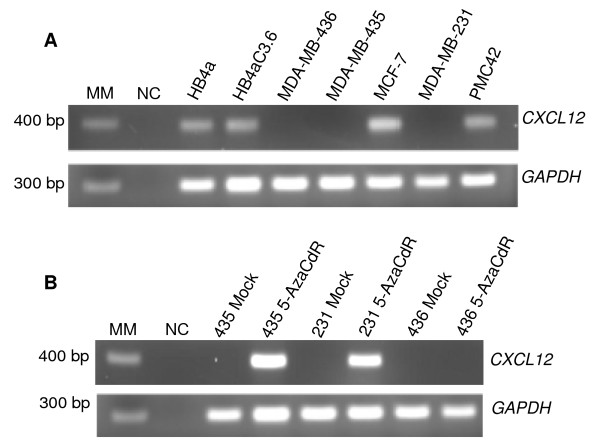
***CXCL12 *expression in breast normal and tumour cell lines and expression after 5-aza-2'-deoxycytidine (5-aza-CdR) treatment**. **(A) **The bands represent positive results for *CXCL12 *expression (383 bps) and *GAPDH *gene was used as housekeeping control (296 bps). **(B) ***CXCL12 *expression evaluation after 5-aza-CdR treatment in MDA-MB-435, MDA-MB-436 and MDA-MB-231 cell lines. The cell lines named Mock were not treated, and those named 5-aza-CdR were treated showing the re-expression of *CXCL12 *on these cell lines. H_2_O was used as negative control and MM 100 bp was used as a molecular weight marker.

We first analysed the *CXCL12 *expression pattern in seven breast tumour cell lines using RT-PCR. A transcript of 383 bps corresponding to the *CXCL12 *gene was detected in the less aggressive carcinoma cell lines MCF-7 and PMC42, in the normal cell line HB4a and in the modified normal cell line HB4aC3.6 with *ERBB2 *overexpression (Figure 2A). In contrast, no detectable levels of *CXCL12 *expression were observed in the highly aggressive tumour cell lines MDA-MB-435, MDA-MB-436 or MDA-MB-231. To definitively determine if *CXCL12 *expression was, in fact, lost, all analyses were repeated at least twice. *GAPDH *expression was detected in all samples (Figure 2A). These data are in agreement with the literature [6], except in the case of the normal cell lines and the tumour cell lines MDA-MB-436 and PMC42, where such data have not been previously described.

### CXCL12 silencing by DNA methylation

To confirm the epigenetic transcriptional silencing of *CXCL12*, we treated the MDA-MB-435, MDA-MB-436 and MDA-MB-231 breast tumour cell lines with the demethylating agent 5-aza-2'-deoxycytidine (5-aza-CdR). The expression of *CXCL12 *was restored in MDA-MB-435 cells upon treatment as previously observed [6]. Expression of *CXCL12 *was also restored in MDA-MB-231 cells, but not in MDA-MB-436 cells (Figure 2B).

The CpGPlot program analysis of 5677 bps of the 5' region of the *CXCL12 *gene identified five putative islands in the *CXCL12 *promoter region. The CpG island is positioned in an area extending from -2594 to +853 (Figure 1A). In this region, five distinct CpG-rich regions were identified in the positions +512 to +853 (island 1), -840 to +506 (island 2), -1118 to -893 (island 3), -1872 to -1575 (island 4) and -2594 to -2323 (island 5). The region comprised of island 2 studied in the breast and colon carcinomas [6,7] was -493 to +168 relative to the TSS and promoter activity was found in the region from -1010 to +122 [22]. The search for transcription factor binding sites by computational analysis revealed eight oestrogen responsive element (ERE) consensus sites, which are defined as 13 bps perfect palindromic inverted repeats with a 3 bps spacing of variable bases (n) 5'GGTCAnnnTGACC 3' [23]. Five of these sites are very distant from the TSS, two are inside island 2 and the essential GACC sequence is absent. Only one site with the sequence aGctgctggGACC (capital letters for those that match with the consensus), which lies 19 bps away from island 4, might be an oestrogen-binding site (Figure 1B). Therefore, we complemented our tumour sample analysis with MSP for *CXCL12 *CpG island 4.

Sodium bisulphite sequencing was carried out on a 297 bps DNA fragment containing 27 CpG dinucleotides (-1872 to -1575) (Figure 1B). We analysed the methylation pattern of eight independent alleles (eight clones) from HB4a and HB4aC3.6 normal cell lines, as well as the PMC42, MCF-7, MDA-MB-436, MDA-MB-435, MDA-MB-231 breast tumour cell lines. The methylation pattern varied among the different cell lines as shown in Figure 1C. The *CXCL12*-negative cell lines, MDA-MB-436 (71%) MDA-MB435 (84%) and MDA-MB-231 (78%) showed CpG dinucleotide hypermethylation. In contrast, the *CXCL12*-positive cell lines, HB4a (11%), HB4aC3.6 (4%), PMC42 (12%) and MCF-7 (5%), had lower levels of CpG dinucleotide methylation (Figure 1C). These results were in agreement with our expression analysis (Figure 2A). On the other hand, the breast tumour cell line MDA-MB-436 had a dense area of methylated DNA and did not regain expression of the *CXCL12 *gene when cultured with the demethylating agent 5-aza-CdR (Figure 2B). The importance of DNA methylation in cancer has been well established [24], and the focus in the field is now changing to include the mechanisms by which other chromatin modifications play a role in cancer development. Among these changes are the covalent modifications of histones that can control gene activity. Histone acetylation and methylation of specific lysine residues, such as lysine 9 in histone H3 or lysine 27 in histone H3, clearly participate in the silencing of genes [25].

We also evaluated the CpG dinucleotide sequence for island 4 in the primary breast tumour samples 1, 3 and 8 (Figure 1D). These samples were chosen from the MSP data analysis, and we have confirmed the pattern that was described.

### MSP analysis in breast tumour cell lines

**Figure 3:**
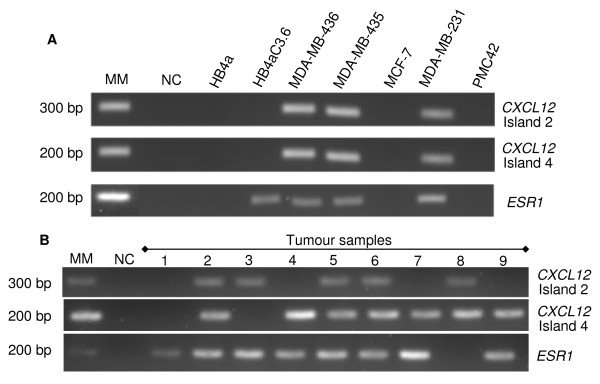
***CXCL12 *and *ESR1 *methylation analyses of breast normal and tumour cell lines and MSP in primary breast tumours**. **(A) **MSP was performed using bisulphite treated DNA with primers that recognize methylated promoter only. The bands represent positive results for the methylation in the islands 2 and 4 for *CXCL12 *and *ESR1 *genes. H_2_O was used as template in the negative control (NC). **(B) **Nine of sixty-nine representative samples are included in the figure. Tumour samples were subjected to the MSP reaction, confirming the standardization done with the cell lines for the methylated condition. The results for *ESR1 *showed promoter methylation in the samples 1 to 7 and 9. The tumour 1 presented both islands negative for methylation of *CXCL12 *gene; however *ESR1 *methylation was present even thought this sample is ERα positive. The tumour 8 represented a sample with unmethylated *ESR1*. The lanes 1, 3 and 8 were representative sequenced samples after bisulphite treatment (see Figure 1D).

Dinucleotides 4 and 5, and 21-24, which were chosen for the design of methylation-specific PCR (MSP) primers, lie within a region that is differentially methylated (Figure 1C). The MSP technique was tested with *CXCL12 *(island 2 and island 4) and *ESR1 *genes in DNA from the tumour cell lines in order to confirm the results from RT-PCR and data from the literature (*ESR1*). The *CXCL12 *MSP results from breast tumour cell lines were identical for the two islands (Figure 3A). The MDA-MB-436, MDA-MB-435 and MDA-MB-231 breast tumour cell lines showed methylation of the two islands, which is in agreement with the observed lack of *CXCL12 *expression. In contrast, the HB4a, HB4aC3.6, MCF-7 and PMC42 cell lines expressing *CXCL12 *showed no methylated bands (Figure 3A).

The *ESR1 *MSP results showed hypermethylation in the MDA-MB-436, MDA-MB-435 and MDA-MB-231 breast tumour cell lines as well as in the HB4aC3.6 normal cell line. The HB4a normal cell line and the MCF-7 and PMC42 breast tumour cell lines did not show any methylated bands (Figure 3A). These data corroborate the literature [10,26] except in the case of cell lines MDA-MB-436, PMC42 and HB4aC3.6, which have not been previously described.

In this work, we did not evaluate normal breast tissue, but Zhou et al. [27] used twenty normal samples and all of them showed *CXCL12 *expression without methylation, which is in agreement with a possible tumoral methylation specificity observed in our results.

### MSP analysis in primary breast tumours

The MSP assay was subsequently used to analyse primary breast tumour samples. All samples showed the band corresponding to the unmethylated state for islands 2 and 4 (data not shown). Based on these results, we can conclude that the samples have cellular heterogeneity due to the presence of normal tissue or infiltrating lymphocytes.

For the methylated condition, nine representative tumour samples are shown (Figure 3B). Of all the samples tested (69), only four were methylated at both islands, and three of these samples methylated at both genes (Figure 3B). Moreover eighteen samples were methylated in both, *CXCL12 *island 4 and *ESR1 *genes (*p *= 0.0220). This finding indicates a strong correlation between the promoter methylation of *ESR1 *and *CXCL12 *island 4. In other words, methylation of the *ESR1 *promoter could be involved in directing the methylation of island 4 (see more details in discussion).

We also wanted to confirm that the presence or absence of island 2 or island 4 methylation observed in MSP (Figure 3B) was not just a PCR artefact. Therefore, we sequenced island 4 isolated from tumour samples 1, 3 and 8 (representative for banding pattern). Island 4 displayed 6% methylation in samples 1 and 3, and 89% methylation in sample 8 (Figure 1D). These results are in agreement with the PCR bands observed in the MSP.

### The correlation between CXCL12 and ESR1 promoter methylation status with patient clinicopathological data

**Figure 4:**
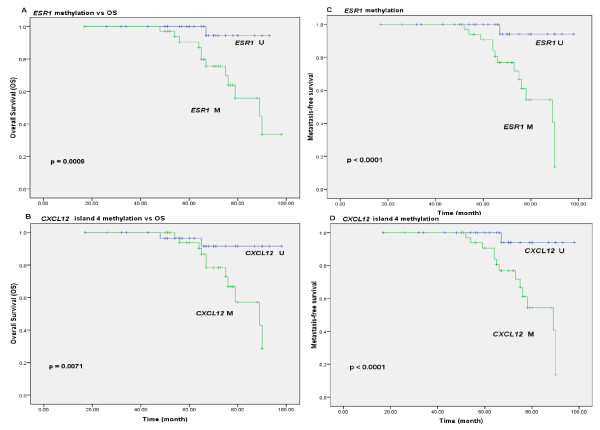
**Kaplan-Meier curves for time to breast cancer progression according to *CXCL12 *methylation status**. Kaplan-Meier estimates are shown for overall survival using **(A) ***ESR1 *methylation, **(B) ***CXCL12 *island 4 methylation, and for metastasis-free survival using **(C) ***ESR1 *methylation, (**D**) *CXCL12 *island 4 methylation. Symbols on the graph lines represent censored data; *p *values are given for log rank tests.

The correlation between the CpG islands of *CXCL12 *and *ESR1 *methylation and the clinicopathological parameters of our samples are shown in Table 1. For island 2, *CXCL12 *methylation was observed in ten of 69 (14.5%) samples and was not significantly associated with any clinicopathological data (Table 1a). For island 4, *CXCL12 *methylation was observed in 37 of 69 (54%) samples. The clinicopathological parameters are shown in Table 1b and hypermethylation in this region was significantly associated with histological grade SBR (*p *= 0.0180), metastasis (*p *= 0.0008) and death (*p *= 0.0019). These results suggested that CpG island 4 might be important as a factor for poor prognosis of disease. In order to test this hypothesis we evaluated all of the clinicopathological data for their prognostic value in a univariate analysis for overall survival (OS) and metastasis-free survival (MFS) using Kaplan-Meier analysis (*p *value for log rank test). The overall survival (OS) was significantly worse for many clinicopathological parameters, which are shown in additional file 1. The graphical results for OS and MFS are shown in Figure 4 for *ESR1 *and *CXCL12*. The OS correlated with *ESR1 *(*p *= 0.0009) and *CXCL12 *island 4 (*p *= 0.0071) methylation (Figure 4A and 4B respectively). The metastasis-free survival correlated with *ESR1 *and *CXCL12 *island 4 (for both, *p *< 0.0001) (Figure 4C and 4D, respectively). The Kaplan-Meier analysis showed that the overall survival and metastasis-free survival were significantly shorter when silencing of *CXCL12 *island 4 and *ESR1 *occurred by gene hypermethylation. The effects of the covariables on OS and MFS were examined in the Cox proportional hazard regression model. The results for the univariate analysis are shown in Table 3. In the multivariate analysis, all variables presenting *p *< 0.2 from the univariate analysis were selected to build the multiple model (Table 3). For overall survival, the presence of metastasis (*p *= 0.0022) was considered a prognostic factor and *ESR1 *hypermethylation showed a statistical trend (*p = *0.0555). Besides ERα, the hypermethylation of *CXCL12 *island 4 and *ESR1 *genes (*p *= 0.0302, *p = *0.0089 and *p *= 0.0046, respectively) was considered an independent prognostic factor for metastasis-free survival. Patients with ERα protein present had the lowest risk of metastasis development (HR 0.3343; 95% CI 0.1247-0.8963). Otherwise, patients with methylation at both *CXCL12 *island 4 and *ESR1 *genes had a risk of developing metastasis, with *CXCL12 *island 4 having the highest risk (HR 7.1355; 95% CI 1.6480-30.8958).

**Table 3:** Time to breast cancer progression in relation to clinicopathological characteristics: Cox proportional hazards model

Analysis	Overall survival			Metastasis-free survival		
	
	HR	95% CI	*p *value	HR	95% CI	*p *value
**Univariate**						
Age	1.0074	0.9717 to 1.0444	0.6901	1.0134	0.9791 to 1.0490	0.4507
Stage	2.8065	1.1063 to 7.1200	**0.0306**	3.3893	1.4386 to 7.9850	**0.0054**
Grade	8.3473	1.1141 to 62.5437	**0.0399**	10.0599	1.3565 to 74.6075	**0.0246**
Tumour size	2.8301	1.0906 to 7.3445	**0.0333**	2.8825	1.1909 to 6.9770	**0.0195**
Lymph node	2.1072	0.9382 to 4.7326	0.0724	1.8045	0.8696 to 3.7445	0.1148
ERα	0.3322	0.1191 to 0.9265	**0.0361**	0.2413	0.0971 to 0.5992	**0.0023**
HER2	0.6813	0.3044 to 1.5247	0.3529	0.8075	0.4005 to 1.6280	0.5521
PR	0.727	0.3487 to 1.5159	0.3976	0.527	0.2614 to 1.0627	0.0749
Recurrence	2.0282	0.7885 to 5.2175	0.1444	1.7645	0.9107 to 3.4189	0.0940
Metastasis	7.824	2.7957 to 21.8962	**< 0.0001**			
Death				2.4134	1.3737 to 4.2402	**0.0022**
Histological type	0.9002	0.2937 to 2.7597	0.8549	0.6848	0.2292 to 2.0455	0.4998
*CXCL12 *Island 4	4.1124	1.3539 to 12.4913	**0.0130**	11.5151	2.6886 to 49.3180	**0.0010**
*CXCL12 *Island 2	0.8083	0.2308 to 2.8313	0.7407	0.7528	0.2207 to 2.5676	0.6518
*ESR1*	3.2917	1.6312 to 6.6424	**0.0009**	3.1300	1.6632 to 5.8904	**0.0004**
**Multivariate**						
Metastasis	5.2652	1.8200 to 15.2323	**0.0022**			
*ESR1*	2.5752	0.9827 to 6.7484	**0.0555**	2.7569	1.0204 to 7.4486	**0.0466**
*CXCL12 *Island 4	*			7.1355	1.6480 to 30.8958	**0.0089**
ERα	*			0.3343	0.1247 to 0.8963	**0.0302**
Tumour size	*			1.9577	0.7137 to 5.3701	0.1942

Abbreviations: HR, hazard ration; CI, confidence interval; SBR, Scarff-Bloom-Richardson classification; ERα, estrogen receptor alpha; PR, progesterone receptor; HER2, Human Epidermal growth factor Receptor-type 2. Statistical significance are in bold. * Variables that were not significant at Cox regression analysis.

**Figure 5:**
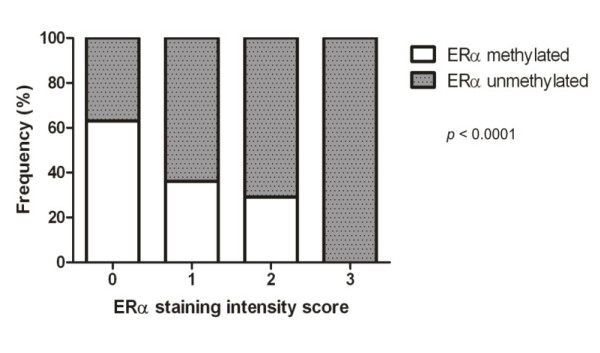
**Correlation of *ESR1 *methylation and protein score staining in the immunohistochemical analysis**. The statistical data showed the correlation between intensity of ERα staining by IHC and frequency of *ESR1 *promoter methylation. The dark bars denote methylated samples and grey bars unmethylated samples. The samples with ERα expression (score 0 to 3, see material and methods) that showed methylation were: scored 0 - 25 with 15 methylated (60%); score 1 - 22 with 8 methylated (36%); score 2 - 16 with 5 methylated (31%), score 3 - 6 samples without methylation. These results show the percentage of samples that were significant with *p *< 0.0001.

*ESR1*methylation was observed in 28 of 69 (41%) tumour samples (Table 1c) and was not significantly associated with age, HER2 or progesterone receptor status, recurrence or histological type. On the other hand, the stage (*p *= 0.0003), tumour size (*p *= 0.0029), histological grade SBR (*p *= 0.0011), lymph node status (p = 0.0288), oestrogen receptor status (*p *= 0.0054), metastasis (*p *< 0.0001) and death (*p *= 0.0105) were significantly associated with *ESR1 *gene methylation. Statistical analyses demonstrated a correlation between the intensity of ERα IHC staining and the frequency of *ESR1 *promoter methylation. Nine out of eleven (82%) ERα-negative cases were hypermethylated. Based on the results from the immunohistochemical analyses, it should be noted that of the samples scored as 0, 15 out of 25 (60%) were methylated; of the samples scored as 1, 8 of the 22 (36%) were methylated; of the samples scored as 2, 5 of the 16 (31%) were methylated; and of the samples scored as 3, none of the six samples were methylated (Figure 5). The number of unmethylated and methylated samples for the scores were submitted to a statistical Fisher's exact test, and statistical significance (*p *< 0.0001) was obtained (Figure 5). This result with ERα-negative status could be due to many mechanisms, including epigenetic mechanisms such as DNA methylation silencing.

These results suggest that in breast cancer patients, silencing of the *CXCL12 *island 4 and *ESR1 *genes by promoter hypermethylation can be used as a complementary marker for metastasis risk assessment.

## Discussion

Breast cancer development and progression is influenced by intrinsic properties of the tumour cells, as well as macro-environmental factors. Extensive interplay exists between the tumour cells and signalling molecules such as chemokines. Muller et al. [3] showed that cancer metastasis is a non-random process; organ selectivity by the tumour cells is largely determined by factors that are expressed in remote organs that eventually turn into preferred sites of metastases. Many reports have demonstrated that chemokines are essential factors for the invasion and survival of tumours [2]. However, many other factors, such as the microenvironment of the breast cancer cells and genetic and epigenetic alterations, might be involved. Epigenetic events in tumours cells have the ability to modulate gene expression and the role of epigenetic alterations in cancer progression has been the focus of increasing interest in recent years [24].

In this study, we assessed the epigenetic regulation of the *CXCL12 *and *ESR1*genes in breast cancer samples from Brazilian women. The regulation of *CXCL12 *expression by promoter hypermethylation is common in colon carcinoma [7] and breast cancer, suggesting that tumour cells that silence *CXCL12 *are at a selective advantage for metastasis [6,27]. In another recent study, positive correlations between *CXCL12 *hypermethylation and ERα-negative status were reported [27]. One of our objectives was to verify in our samples the methylation findings obtained in other female populations. We evaluated two CpG islands of the *CXCL12 *gene. The first (island 2) was hypermethylated in ten of 69 (14.5%) tumour samples. This region had already been analysed by another group that found DNA methylation in five of 15 (33.3%) American breast cancer samples [6]. In another study [25] with patient samples from a Chinese population, it was verified that 33 of 63 (52.4%) tumour samples were hypermethylated in the same region. These different results might be due to population or environmental differences. The results for island 2 (Table 1a) showed that no statistical significance was identified for any of the variables. We still do not know if different populations might possess promoter regions that are variable due to SNPs or other mechanisms that can switch genes off.

We have also analysed the *CXCL12 *5' upstream transcription region in more detail and found a second CpG island we refer to here as island 4, which is near a DNA consensus site for transcriptional activation. Island 4 is located 565 bps from the region studied by Garcia-Moruja et al. [22], and we speculate that this region is important for transcriptional regulation. However, Antequera and Bird [28] argue that the importance of CpG islands situated near the TSS is not always obvious, and that new transcripts beginning in CpG islands distant from the main promoter have been found. Island 4 is located 19 bps away from a 5" oestrogen responsive element (ERE) binding site. No data exist to prove that these ERE consensus sequences could be functional. However, Lin et al. [29] demonstrated discernable differences between functional and non-functional ERE sites (1234 sites were found) and the *CXCL12 *gene was one of the oestrogen-responsive genes identified in microarray experiments. Other studies have demonstrated that *CXCL12 *might be a target for oestrogen receptor binding [14,30]. We have concluded that the ERE proximal to island 4 could be involved in the transcriptional regulation of *CXCL12*. This interaction was modelled by Hall and Korack [14], who suggested that oestrogen activates *CXCL12 *expression. In this model, CXCL12 binds to the cell surface receptor, CXCR4, in a potentially autocrine and/or paracrine (MAPK cascade) manner, resulting in the activation of cell proliferation.

The MSP results for island 4 (Table 1b) showed a statistical correlation with three variables related to metastasis, reinforcing a probable role for island 4 in the metastatic process. From these variables, we highlight the correlation of island 4 hypermethylation with *ESR1 *silencing (Table 1c). In order to study this probable correlation, Kaplan-Meier and Cox analyses were performed. These analyses confirm that the silencing of both *CXCL12 *island 4 and *ESR1 *genes by DNA hypermethylation and probably the absence of ERα, are prognostic factors for metastasis-free survival (Table 3).

The results showed that *ESR1 *promoter methylation occurred at a higher frequency in samples with methylated *CXCL12 *island 4 than in samples with methylated island 2 (Figure 3B). In addition, previous studies [9,10,20,31] reported that the *ESR1 *gene possesses a hypermethylated promoter region. This might explain the correlation found in this study between the hypermethylation of *CXCL12 *island 4, but not island 2, and the oestrogen receptor negativity. We speculate that these effects are caused by the proximity of the CpG island to an ERE. Curradi et al. [32] observed that the presence of about eight methylated dinucleotides could inhibit transcription because approximately 700 bps of distance is required for chromatin modifications. Therefore, it is possible that the methylation of island 4 could attenuate or even inactivate ERE binding activity.

On the other hand, several studies have also shown the inverse correlation. The absence of ERα could inactivate target genes that possess an ERE site. It is known that 60% of primary breast tumours are ERα-positive, and two-thirds of the advanced tumours do not respond to therapy with anti-estrogens such as tamoxifen (Novaldex^®^). However, more than a third of patients do not express ERα at the time of diagnosis, and a fraction of tumours that are positive at the time of diagnosis often lose ERα expression [9]. Breast tumour cells in which ERα is absent cannot be regulated by oestrogen, and endocrine therapy is not an option, resulting in a poorer prognosis. Lapidus et al. [33] showed for the first time that *ESR1 *is inactivated by CpG island DNA methylation in cell lines and primary breast tumours. The same group [31] also used MSP to show that breast cancers expressing ERα had an unmethylated promoter region. Mirza et al. [10] and Zhao et al. [11] detected 66% and 60% *ESR1 *hypermethylation in Indian and Chinese populations, respectively. Thus, the hypermethylation of ERα is commonly seen in several populations and seems to be relevant to hormonal treatment response. According to this, Leu et al. [34] proposed a model suggesting that the progesterone receptor gene (*PGR*) or another hypothetical target containing an ERE consensus site inside the promoter region becomes hypermethylated and silenced by repressor proteins when ERα is not expressed. In other words, *ESR1 *inactivation and the consequent lack of ERα cause all target genes to become susceptible to epigenetic silencing. Apparently, *ESR1 *gene silencing by promoter hypermethylation and the consequent absence or decrease of ERα expression are able to lead to *CXCL12 *gene silencing by hypermethylation.

Consequently, the absence of CXCL12 signals the cells to form metastasis in tumours with a high tumour grade with negative ERα status, increasing the probability of patient death. We do not yet know if island 4 hypermethylation could be involved in the chromatin alterations associated with the blockage of ERα binding, or if the absence of ERα due to epigenetic silencing can contribute to the hypermethylation of C*XCL12*.

The results presented here show that epigenetic alterations might play an important role in the downregulation of CXCL12 mRNA in breast cancers in Brazilian women. Our results, together with recent findings, emphasise the importance of the CXCL12/CXCR4 signalling axis in the organ-specific patterns of metastasis. Epigenetic events could regulate other genes involved in the development of breast cancer and could also be used to predict a better prognosis. The results presented here could also be interpreted as a cause of the eventual resistance seen in response to endocrine therapy.

## Conclusions

Our results have shown, for the first time, a correlation between hypermethylation of *CXCL12 *island 4 and *ESR1 *in patients with histologically advanced cancer, the presence of metastases and death. This correlation could be an important factor in prognosis, cancer prevention and treatment of these patients.

## Competing interests

The authors declare that they have no competing interests.

## Authors' contributions

EASR, carried out the experimental data acquisition, performed data analyses and interpretation and drafted the manuscript. AAC critically revised the manuscript. RS was an undergraduated student and helped with sequencing data collection. KB was the biostatistician in the study. EMSFR and IJC provided patient material and clinicopathological data and critically revised the manuscript. FOP critically revised the manuscript and gave equipment support. EMS critically revised the manuscript and suggested experiments. FFC helped in the experimental design and critically revised the manuscript. GK designed and coordinated the study, supplied administrative support and critically revised the manuscript. All authors read and approved the final manuscript.

## Pre-publication history

The pre-publication history for this paper can be accessed here:

http://www.biomedcentral.com/1471-2407/10/23/prepub

## Supplementary Material

Additional file 1 **Overall (OS) and metastasis-free survival (MFS) probabilities calculated by Kaplan-Meier estimates (*p *value for log rank test)**. Kaplan-Meier results for samples. Click here for file
