# Supplementary material for: Simultaneous CXCL12 and ESR1 CpG island hypermethylation correlates with poor prognosis in sporadic breast cancer
Source: BMC Cancer. 2010 Jan 28;10:23. doi: 10.1186/1471-2407-10-23 (PMC2834618; doi:10.1186/1471-2407-10-23)
Supplement: Additional file 1 — Overall (OS) and metastasis-free survival (MFS) probabilities calculated by Kaplan-Meier estimates (p value for log rank test). Kaplan-Meier results for samples. [file 1471-2407-10-23-S1.DOC]

**Additional files**

Overall (OS) and metastasis-free survival (MFS) probabilities as calculated by Kaplan-Meier estimates (*p* value for log rank test).

| **Characteristic** | **OS**  ***p* value** | **MFS**  ***p* value** |
| --- | --- | --- |
| Stage | **0.0237** | **0.0031** |
| Grade | **0.0138** | **0.0054** |
| Tumour size | **0.0026** | **0.0143** |
| Lymph node | **0.0154** | **0.0241** |
| ERα | 0.0937 | **0.0045** |
| PR | 0.6895 | 0.1943 |
| HER2 | 0.3700 | 0.2264 |
| Recurrence | 0.6962 | **0.0204** |
| Metastasis | **< 0.0001** |  |
| Death |  | **< 0.0001** |
| Histological type | 0.8543 | 0.4953 |
| *CXCL12* Island 4 | **0.0071** | **< 0.0001** |
| *CXCL12* Island 2 | 0.7396 | 0.6493 |
| *ESR1* methylation | **0.0009** | **< 0.0001** |

*p* value from statistical analysis by Kaplan-Meier test.

Statistical significant are in bold
